# 3D printed multi-drug-loaded suppositories for acute severe ulcerative colitis

**DOI:** 10.1016/j.ijpx.2023.100165

**Published:** 2023-01-29

**Authors:** Atheer Awad, Eleanor Hollis, Alvaro Goyanes, Mine Orlu, Simon Gaisford, Abdul W. Basit

**Affiliations:** a Department of Pharmaceutics, UCL School of Pharmacy, University College London, 29-39 Brunswick Square, London WC1N 1AX, UK; b FabRx Ltd., Henwood House, Henwood, Ashford, Kent TN24 8DH, UK; c Departamento de Farmacología, Farmacia y Tecnología Farmacéutica, I+D Farma (GI-1645), Facultad de Farmacia, Materials institute (iMATUS) and Health Research Institute (IDIS), Universidade de Santiago de Compostela, 15782 Santiago de Compostela, Spain

**Keywords:** Inflammatory bowel disease, Rectal corticosteroids, Janus kinase (JAK) inhibitors, Extrusion-assisted three-dimensional printing of drug products, Pressure-assisted micro syringe, Additive manufacturing of pharmaceuticals and medicines, Digital design and manufacture of drug delivery systems

## Abstract

Acute severe ulcerative colitis (ASUC) is a growing health burden that often requires treatment with multiple therapeutic agents. As inflammation is localised in the rectum and colon, local drug delivery using suppositories could improve therapeutic outcomes. Three-dimensional (3D) printing is a novel manufacturing tool that permits the combination of multiple drugs in personalised dosage forms, created based on each patient's disease condition. This study, for the first time, demonstrates the feasibility of producing 3D printed suppositories with two anti-inflammatory agents, budesonide and tofacitinib citrate, for the treatment of ASUC. As both drugs are poorly water-soluble, the suppositories' ability to self-emulsify was exploited to improve their performance. The suppositories were fabricated via semi-solid extrusion (SSE) 3D printing and contained tofacitinib citrate and budesonide in varying doses (10 or 5 mg; 4 or 2 mg, respectively). The suppositories displayed similar dissolution and disintegration behaviours irrespective of their drug content, demonstrating the flexibility of the technology. Overall, this study demonstrates the feasibility of using SSE 3D printing to create multi-drug suppositories for the treatment of ASUC, with the possibility of titrating the drug doses based on the disease progression.

## Graphical abstract

**Figure:**
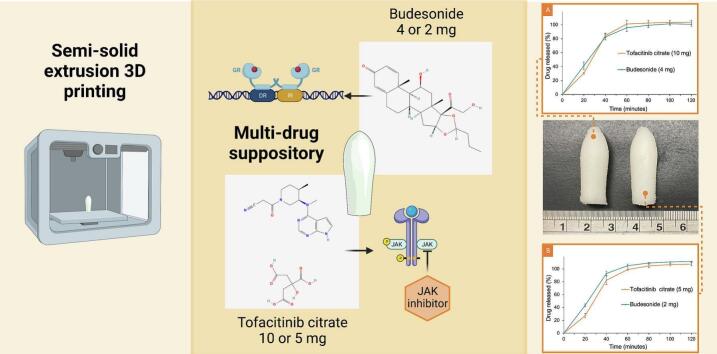

## 1 Introduction

Ulcerative colitis (UC) is a chronic, idiopathic inflammatory disease of the rectum, characterised by diffuse mucosal inflammation, extending variably across the proximal lengths of the colon (Abdalla and Herfarth, 2016). Common symptoms of UC include bloody stool, diarrhoea, incontinence, and fatigue, resulting in life-limiting consequences for the patient (Ungaro et al., 2017). Approximately 15–30% of patients tend to experience at least one episode of acute severe ulcerative colitis (ASUC), a potentially life-threatening medical emergency requiring timely diagnosis and treatment initiation (Rosiou and Selinger, 2021). Intravenous (IV) corticosteroids (e.g., prednisolone) are the first-line medical intervention for ASUC (Kedia et al., 2014). However, in spite of their widespread use, resistance is observed in one third of patients, who subsequently rely on second-line therapeutics including immunosuppressants, biologic agents, and aminosalicylates (Endo et al., 2016). Thus, it has been suggested that patients may respond better to treatments involving the simultaneous administration of two medications, especially if the two agents exhibit their action at different stages of the inflammatory cascade (Judge et al., 2021; Privitera et al., 2021).

As an example, clinical outcomes could be improved by combining budesonide, a second-generation glucocorticoid (Pastorelli et al., 2020), with tofacitinib citrate, a novel, small-molecule Janus-associated kinase (JAK) inhibitor (Cada et al., 2013). Budesonide's anti-inflammatory mechanism lies in its ability to bind to glucocorticoid-receptors (GR), resulting in the genes' suppression and inhibiting transcription factors that control pro-inflammatory mediators (Williams, 2018). Tofacitinib citrate on the other hand competitively binds to the enzymes JAK 1 and JAK 3, resulting in the downregulation of signal transducers and activators of transcription that consequently reduce interleukin levels (Cada et al., 2013). As both drugs have different mechanisms of actions, their concurrent administration could potentially result in a synergistic effect, marked by the improved management of extraintestinal manifestations of IBD and reduced risks of treatment failure.

Oral and IV delivery of drugs in ASUC is challenging, possibly resulting in systemic side effects and low dose availability at the disease site, limiting their clinical effectiveness (Hua, 2020). Additionally, oral drugs targeting the colon must traverse the whole alimentary canal before arriving at the disease site, causing a delay in the therapeutic response (Awad et al., 2022; McCoubrey et al., 2023). In contrast, a rectal formulation offers a practical alternative to enhance therapeutic efficacy, maximising drug concentrations at the disease site, while lowering systemic side effects (Hua, 2019). This type of formulation can also be self-administered by the patient, unlike parenteral formulations which require medically trained personnel.

Thus, the aim of this work was to fabricate suppositories loaded with budesonide and tofacitinib citrate for the treatment of ASUC. In current practice, the compounding of suppositories most commonly involves fusion moulding, which is a lengthy process that requires several steps (Jannin et al., 2014). Three-dimensional (3D) printing is a novel manufacturing approach that has shown potential in overcoming the limitations of conventional production methods (Tagami et al., 2020; Xu et al., 2021). Semi-solid extrusion (SSE) 3D printing allows for drugs and lipid excipients to be directly combined and simplifies the drug-loading process (Chatzitaki et al., 2021; Seoane-Viaño et al., 2020a; Seoane-Viaño et al., 2020b), making it well suited for this approach.

As both tofacitinib citrate and budesonide suffer from poor water solubilities (Ali et al., 2010; Salimi et al., 2014), their formulation into self-emulsifying drug delivery systems (SEDDSs) could improve their solubilities, further enhancing their bioavailability and clinical outcomes (Panigrahi et al., 2018). SEDDSs are lipid-based systems in the form of isotropic blends of oils and surfactants (and occasionally co-surfactants) that result in stable oil-in-water (o/w) fine emulsions (Neslihan Gursoy and Benita, 2004).

Hence, this study reports the fabrication of multi-drug suppositories using SSE 3D printing. To assess the feasibility of this approach, the suppositories underwent characterisation to determine their *in vitro* performance including disintegration and self-emulsifying time, drug loading, droplet size and zeta (ζ) potential.

## 2 Materials and methods

### 2.1 Materials

Tofacitinib citrate (MedChemTronica, Sweden) and budesonide (Fisher Scientific, New Hampshire, USA) were used as the active pharmaceutical ingredients (APIs). The main suppository base was comprised of Gelucire® 44/14 (Gattefossé, France). Coconut oil from *Cocos nucifera* (Sigma-Aldrich, Germany) and *N*,*N*-Dimethylacetamide (N,N-DMA; Thermo scientific, Germany) were incorporated into the formulation as an additional excipients to improve its printability, with the latter also improving the solubilities of tofacitinib citrate and budesonide.

### 2.2 Semi-solid extrusion 3D printing

Pre-selected proportions of lipid excipients, co-solvent, and drugs were weighed in preparation for compounding of the formulations (Table 1). Each TOF10-BUD4 suppository contained 10 mg tofacitinib citrate and 4 mg budesonide, whereas each TOF5-BUD2 suppository contained 5 mg tofacitinib citrate and 2 mg budesonide. 4 and 2 mg budesonide dosing in suppositories has been previously reported in the literature (Kruis et al., 2019; Kruis et al., 2022). Tofacitinib citrate is currently available in 2 oral doses; 10 and 5 mg. As there are currently no rectal forms of tofacitinib citrate commercially available (Electronic Medicines Compendium, 2022), its UC oral doses were adopted in this study.

**Table 1:** Summary of the compositions of the formulations used for printing the suppositories.

Formulation	Tofacitinib citrate (%*w*/w)	Budesonide (%w/w)	Gelucire® 44/14 (%w/w)	Coconut oil (%w/w)	N,N-DMA (%w/w)
TOF10-BUD4	0.49	0.19	75.46	18.86	5.00
TOF5-BUD2	0.24	0.10	75.73	18.93	5.00

Selected ratios of excipients were melted at 75 °C in a 100 mL glass beaker, positioned on a Super-Nuova Multi-Position Digital Stirring Hotplate (Thermo Fisher Scientific, Massachusetts, USA) under magnetic stirring (500 rpm). Once the mixture was completely melted, selected amounts of the drugs were added, whilst keeping the mixture under magnetic stirring until complete solubilisation of the drugs was achieved. Upon solubilisation, the formulation was directly transferred to a 20 mL Injekt® disposable syringe (Braun, Germany) and placed in a −20 °C freezer to solidify. Once solid, a tapered 0.024″ - 0.61 mm extrusion tip (Fisnar, UK) was attached to the end of the syringe which was then positioned into the SSE printhead of the M3DIMAKER 2 3D printer (FabRx Ltd., UK). Previously prepared 3D models (Dimensions: 12 mm × 36 mm) of the suppositories were transformed into .gcode files by means of Cura software (v 15.04.6, Ultimaker Utrecht, Netherlands) and printed using the following parameters: 29 °C printing temperature; 0.5 mm layer height; 2.4 mm shell thickness; 25 mm/s flow speed. As the printing temperature was close to room temperature, an evaporative air cooler (Igenix, UK) was placed in front of the 3D printer during the printing process and set at fan speed 3 to ensure printing consistency despite changes in the atmospheric temperature. Following printing, the 3D printed suppositories were transferred into a 4 °C fridge and allowed to solidify, and subsequently weighed and stored in a −20 °C freezer. This temperature was selected since it is typically used for budesonide's storge, ensuring its stability within the suppositories.

### 2.3 Characterisation of the 3D printed suppositories

#### 2.3.1 Differential scanning calorimetry (DSC)

Differential scanning calorimetry (DSC) was performed to characterise the pure drugs, excipients, and 3D printed suppositories. N,N-DMA was not tested due to safety reasons since its flash point is at 63 °C. DSC measurements were made with a Q2000 DSC (TA instruments, Waters LLC, USA). Calibrations for the cell constant and enthalpy were made with indium (*T*_m_ = 156.6 °C, Δ_f_*H* = 28.71 J/g), in accordance with the specified manufacturer guidelines. Nitrogen was used as the purge gas, at a flow rate of 50 mL/ min, for all the performed experiments. Baseline correction, *T*_m_ and enthalpy values were obtained using the TA Advantage software for Q series (Version 5.5.3, TA instruments, Waters LLC, USA) and examined via TA Instruments Universal Analysis 2000 (Version 4.5.0.5, TA instruments, Waters LLC, USA). Tzero aluminium pans and pin-holed hermetic lids (TA instruments, Waters LLC, USA) were used with an average sample mass of 3–5 mg.

#### 2.3.2 X-ray powder diffraction (XRPD)

Discs of 23 mm diameter x 1 mm height were 3D printed using both formulations and analysed. Samples of the pure drugs and Gelucire® 44/14 were also tested. Due to the low melting temperature of coconut oil (23–27 °C) and liquid form of N,N-DMA it was not possible to analyse them individually and instead, a blank suppository containing Gelucire® 44/14, coconut oil and N,N-DMA at the same ratios used in the formulations was analysed. The X-ray powder diffraction patterns were acquired using a Rigaku MiniFlex 600 (Rigaku, Massachusetts, USA) equipped with a Cu Kα X-ray source (λ = 1.5418 Å) having an intensity of 15 mA and a voltage of 40 kV. The angular range of data acquisition was 3–40° 2θ, with stepwise size of 0.02° set at a speed of 2°/min.

#### 2.3.3 Fourier transform-infrared spectroscopy (FTIR)

A Spectrum 100 FTIR spectrometer (PerkinElmer, Massachusetts, USA) was utilised for the collection of the infrared spectra. Pure drug powders and excipients were measured as references for the analysis of the two suppositories. Each sample was scanned over a range of 4000–650 cm^−1^ at a resolution of 1 cm^−1^ for 64 scans.

#### 2.3.4 Scanning electron microscopy (SEM)

Sections of the 3D printed suppositories were imaged using a Phenom Pro Desktop Scanning Electron Microscope (Thermo Fisher Scientific, UK) at 10 kV accelerating voltage. Each sample was placed on a self-adhesive carbon disc, mounted on a 25 mm aluminium stub, and coated with 25 nm of gold via a sputter coater. An optical navigation camera was used to attain surface images of the suppositories.

#### 2.3.5 Transmission electron microscopy (TEM)

50 mg of each suppository was placed in 100 mL of purified water under magnetic stirring at 100 rpm until the emulsions were formed. Then, a drop of diluted SEDDS was deposited on the carbon-formvar film grid, stained by 1% aqueous solution of phosphotungstic acid and observed after drying. TEM images of the emulsion droplets were captured using a CM120 Philips Biotwin transmission electron microscope (Eindhoven, The Netherlands).

#### 2.3.6 Determination of disintegration time

*In vitro* disintegration testing was conducted in purified water at 37 ± 0.5 °C using a United States Pharmacopeia (USP) disintegration apparatus (ZT43, Copley, UK) with a basket rack assembly and perforated discs. The setup was marginally adjusted to confer with the European Pharmacopeia's (EP) requirements (European Pharmacopeia, 2022; Seoane-Viaño et al., 2020b). The volume of water in the vessel was adjusted as per the USP requirements (i.e., when the basket is raised to its highest point the wire mesh remains at a minimum 15 mm below the surface of the liquid and when lowered it moves down by ≥25 mm from the bottom of the beaker). Each suppository (*n* *=* *3*) was placed on the basket rack assembly and a perforated disc was placed on top of it. Disintegration was regarded as achieved when components of the suppository separated, or upon the suppository's softening and undergoing an appreciable change in its form.

#### 2.3.7 Determination of self-emulsification time

The self-emulsification time of the SEDDS suppositories (*n* *=* *3*) was established using a type II USP dissolution apparatus. A sample from each formulation type was melted in a glass vial at 60 °C using a hotplate. 20 μL of the molten sample was gradually added to 500 mL of purified water at 37 ± 0.5 °C. Gentle agitation was achieved by rotating standard stainless-steel dissolution paddles at a constant speed of 50 rpm. Self-emulsification was recorded as the time taken for a clear solution to form following the addition 20 μL of the molten samples.

#### 2.3.8 Determination of droplet size and ζ potential

An emulsion for each of the formulations (*n* *=* *3*) was prepared by dissolving a 50 mg portion of each suppository in 100 mL of purified water at 37 ± 0.5 °C, with continuous stirring at 100 rpm. Droplet size (i.e., the mean diameter of lipid droplets in the emulsion and polydispersity index) and ζ potential (i.e., the charge of droplets within the emulsion) were established using a Zetasizer Nano (Malvern Instrument Limited, UK).

#### 2.3.9 Drug loading

Each suppository (*n* *=* *3*) was dissolved in 100 mL of 0.1 M phosphate buffer (pH 8.0) at 37 ± 0.5 °C under continuous magnetic stirring at 100 rpm until complete dissolution was achieved. 1 mL aliquots were withdrawn and diluted by half using absolute ethanol. Samples were then filtered via a 0.22 μm syringe filter (Sigma Aldrich, UK). The total amount of drug in each sample was quantified using a Hewlett Packard 1260II Series HPLC system (Agilent Technologies, UK). The assay involved injecting 20 μL of each sample through an Eclipse plus C18 column, 150 mm × 4.6 mm (Zorbax, Agilent Technologies, UK) at 40 °C. The mobile phase was comprised of A) methanol and B) purified water and was pumped at a flow rate of 1 mL/min. The gradient elution was as follows: 50% A from 0 to 4 min, followed by 50–80% A from 4 to 4.10 min, then kept at 80% A from 4.10 to 8 min and finally 80–50% A from 8 to 12 min. The eluents were screened at a wavelength of 254 nm.

#### 2.3.10 In vitro drug release

*In vitro* drug release profiles from the suppositories were acquired by placing each suppository (*n* *=* *3*) in 100 mL of 0.1 M phosphate buffer (pH 8.0), stirred at a constant paddle speed of 100 rpm, and kept at a temperature of 37 ± 0.5 °C. 1 mL aliquots were withdrawn from each sample at pre-selected time points (20, 40, 60, 80, 100, 120 min) and diluted by half with absolute ethanol. Each sample was then filtered via a 0.22 μm syringe filter (Sigma Aldrich, UK) and analysed using the HPLC method described in Section 2.3.9.

The dissolution profiles of both formulations were compared using an ƒ_2_ similarity test to determine if the changes in drug doses still result in similar release profiles or not. The similarity factor ƒ_2_ (i.e., a logarithmic reciprocal square root transformation of the sum of the squared error) was calculated using eq. (1) (Moore and Flanner, 1996).

(1) $$f_{2}=50\times \log\left\{{\left[1+\frac{1}{n}\sum_{t=1}^{n}{\left(R_{t}-T_{t}\right)}^{2}\right]}^{-\frac{1}{2}}\times 100\right\}$$

where R_t_ refers to the release profile of the reference formulation, T_t_ is the release profile of the test formulation, both measured at time point *t*, and *n* refers to the number of dissolution time points being studied (Gohel et al., 2009).

ƒ_2_ values may vary between 0 and 100 and typically, release profiles are considered to be alike when their ƒ_2_ value is over 50 (Shah et al., 1998). An ƒ_2_ value of 100 indicates that the release profiles are identical. Furthermore, the lower an ƒ_2_ value is, the higher is the dissimilarity between the dissolution profiles being compared.

### 2.4 Statistical analysis

One-way analysis of variance (ANOVA) statistical analysis was performed to assess the statistical significance of the differences between the attained results for the different suppository formulations. Statistical analysis between disintegration time, self-emulsifying time tests, drug loading, droplet size and ζ potential were performed. All analyses were completed using Origin (OriginPro 2019, OriginLab corporation, USA), wherein *P* < 0.05 inferred statistically significant differences.

## 3 Results and discussion

In this work, SSE 3D printing was successfully used to fabricate multi-drug suppositories aimed for the treatment of ASUC. Initially, the poor solubilities of tofacitinib citrate and budesonide hindered their miscibility with the suppository base. Thus, to overcome this, a novel approach involving the incorporation of an organic solvent into the printing ink was adopted. Herein, N,N-DMA was selected as tofacitinib citrate has been previously shown to be freely soluble in this solvent (Kumar et al., 2015; Therapeutic Goods Aministration - Australian Government, 2019). This solvent is approved by the United States Food and Drug Administration (FDA) for use in IV injections (Kim, 1988; Weiss et al., 1962) and has displayed a good safety profile (Hempel et al., 2007; Trame et al., 2013), with studies showing a no-observed-adverse-effect level (NOAEL) of 60 mg/kg per day for oral toxicity (European Chemical Agency, 2011).

**Fig. 1:**
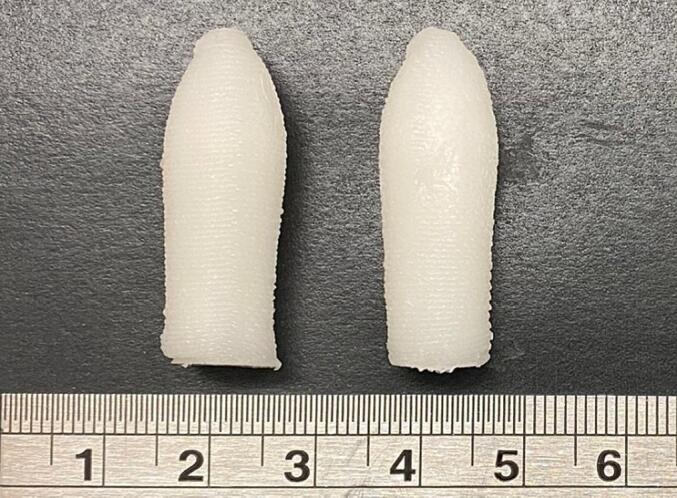
Photograph of the (left) TOF10-BUD4 and (right) TOF5-BUD2 suppositories. Scale shown in cm.

Various concentrations of N,N-DMA were tested (i.e., 1, 3 and 5% *w*/w), wherein 5% w/w was identified as being suitable for dissolving both APIs in their varying doses (Table 1). Both formulations were successfully used for SSE 3D printing and resulted in well-defined suppositories with acceptable consistencies (Fig. 1). Three suppositories were printed per batch, wherein the printing time for one batch was 16 min 30 s. Following printing, the suppositories were directly transferred to a 4 °C fridge to accelerate the solidification process, then packed individually and stored in a −20 °C freezer.

The suppositories were printed vertically, as previous studies revealed that the vertical orientation resulted in a better resolution and more defined shapes (Seoane-Viaño et al., 2020b). As tofacitinib citrate is currently only available in oral dosages (oral solution, film-coated tablets, and prolonged release tablets (Mayo Clinic, 2022)), this work is the first to introduce a tofacitinib citrate dosage form for local drug delivery to the rectum. In this study, a large-sized suppository 3D design was selected to allow for 10 mg tofacitinib citrate and 4 mg budesonide to be concurrently loaded within the same dosage form whilst maintaining adequate consistencies that withstand manual handling. This is because a smaller suppository size would mean that higher drug amounts must be incorporated into the formulations to reach the required doses, which in turn requires a larger volume of N,N-DMA to be dissolved, posing the risk of making the formulations too runny and unsuitable for 3D printing.

The weights of the suppositories ranged between 2.04 g to 2.23 g for the TOF10-BUD4 formulation and 2.01 g to 2.17 g for the TOF5-BUD2 formulation (Table 2), thus, highlighting a reproducible printing process (*P* > 0.05). Compared to theoretical values, the drug loading values of tofacitinib citrate and budesonide in the suppositories were 103.5 ± 2.67% and 101.5 ± 2.02%, and 107.43 ± 2.51% and 113.83 ± 5.98% for the TOF10-BUD4 and TOF5-BUD2 formulations, respectively (Table 2). These values indicate that the two APIs did not undergo thermal degradation during formulation preparation or printing. This was expected since thermal studies using thermogravimetric analysis showed that both APIs can withstand heating up to ∼200 °C (Appendix A Supplementary data). The difference between the actual and theoretical drug loading values in both the formulations is hypothesised to be a result of the N,N-DMA evaporation during formulation compounding and/or 3D printing process.

**Table 2:** Characterisation of the of the 3D printed suppositories (mean ± SD).

Formulation	Weight (g)	Tofacitinib citrate dose (mg)	Budesonide dose (mg)	Tofacitinib citrate loading (%)	Budesonide loading (%)
TOF10-BUD4	2.12 ± 0.06	10.83 ± 0.23	4.24 ± 0.06	0.50 ± 0.01	0.20 ± 0.004
TOF5-BUD2	2.09 ± 0.07	5.48 ± 0.20	2.39 ± 0.10	0.26 ± 0.01	0.11 ± 0.01

**Fig. 2:**
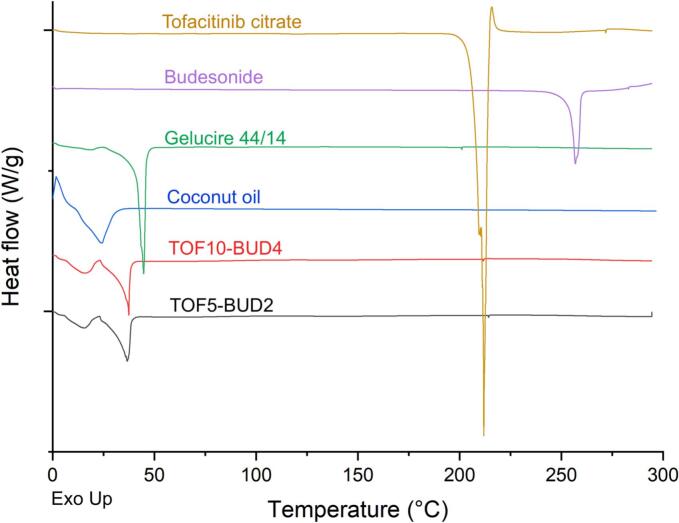
Differential scanning calorimetry (DSC) thermograms of the pure drugs, excipients and 3D printed suppositories.

**Fig. 3:**
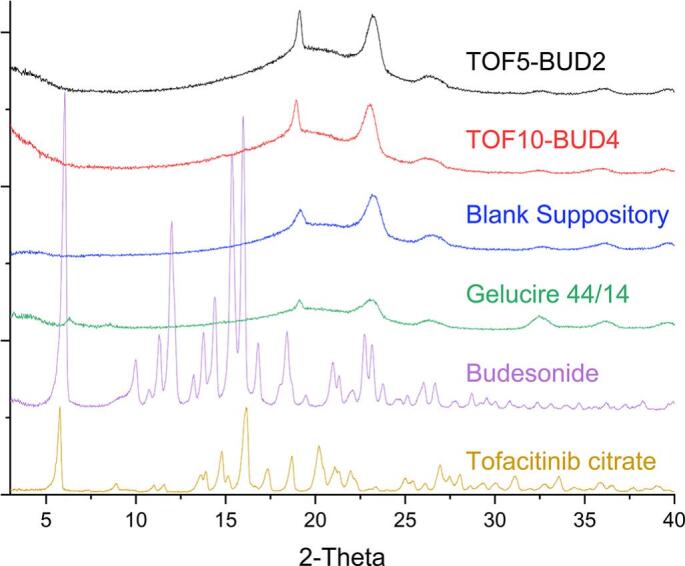
X-ray diffractograms of the pure drugs, lipid base, blank suppository and drug-loaded suppositories.

DSC and XRPD were performed to characterise the physical state of the raw APIs and excipients before printing and within the final dosage forms. DSC thermographs showed that the lipid excipients Gelucire® 44/14 and coconut oil have melting points of approximately 45 °C and 25 °C, respectively (Fig. 2). Correspondingly, the melting peaks of both lipid excipients were present in the thermographs of the suppositories. The melting points of these lipid bases suggest that the suppositories will melt at body temperature (∼37 °C) following their insertion into the rectum, releasing the two drugs locally into the disease site. Pure tofacitinib citrate shows a melting endotherm at 218 °C followed by a recrystallisation exotherm at 248 °C. Budesonide on the other hand shows a melting endotherm at 260 °C. Nonetheless, no peaks corresponding to either of the drugs were observed in the suppositories, indicating that both APIs are either molecularly dispersed within the lipid excipients or dissolved within them as the temperature increases during the DSC process. Corroborating with the DSC data, no crystalline peaks corresponding to tofacitinib citrate and budesonide were observed in the X-ray diffractograms of the suppositories (Fig. 3).

**Fig. 4:**
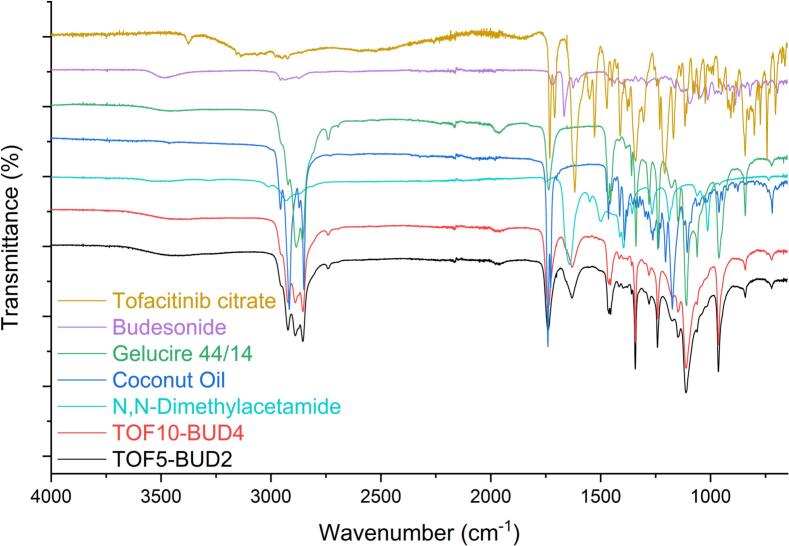
FTIR spectra of the pure drugs (tofacitinib citrate and budesonide), the lipid excipients (Gelucire® 44/14 and coconut oil), N,N-DMA and the suppositories (TOF10-BUD4 and TOF5-BUD2).

FTIR spectroscopy was performed on the suppositories to examine the possibility of interactions occurring between the different drugs and/or compounds within the suppositories (Fig. 4). The FTIR patterns of TOF10-BUD4 and TOF5-BUD2 suppositories indicate comparable formulation compositions. This is to be expected as the formulations differ marginally in the doses of APIs (Table 1). However, because of the low concentrations of APIs within the formulations, the peaks corresponding to the APIs were not distinguishable in the FTIR spectra of the suppositories.

**Fig. 5:**
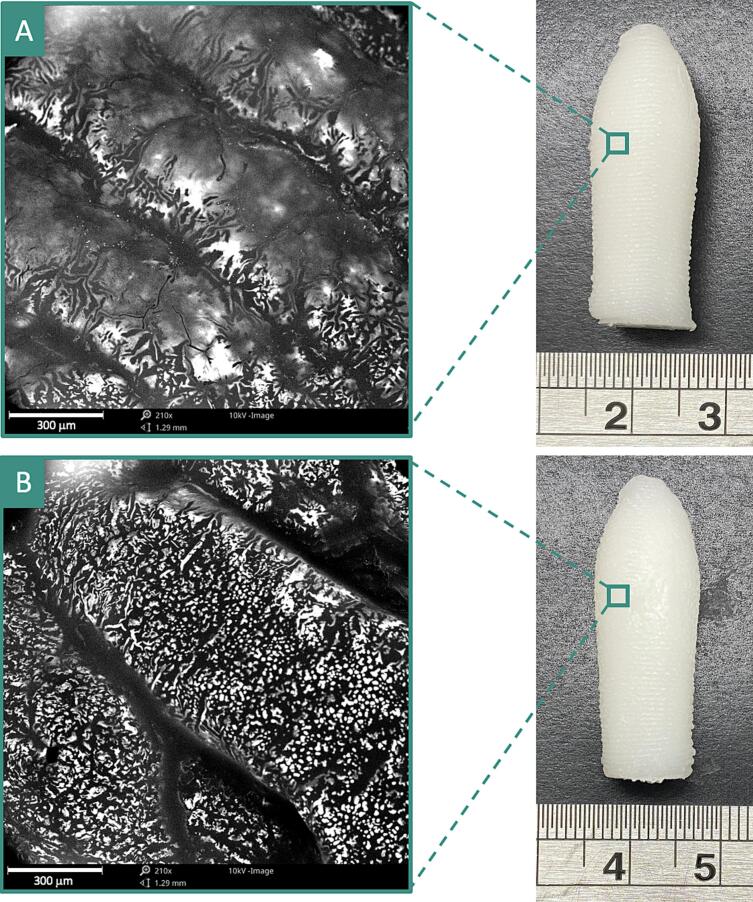
(left) SEM images and (right) photographs of the (A) TOF10-BUD4 and (B) TOF5-BUD2 suppositories. Scale shown in the photographs is in cm.

Photographs and SEM images of the surface of the suppositories indicate that the layers are well connected to one another, forming well-defined, solid suppositories with good structural integrity (Fig. 5). The printed layers were able to support sequential layers during the entire printing process, enabling appropriate and reproducible printing of the suppositories.

Disintegration time of the suppositories evaluated the time taken for mechanical breakdown of the suppository into small parts or a considerable change in its appearance (Daniela Amaral Silva et al., 2018). The TOF10-BUD4 formulation underwent disintegration faster compared to the TOF5-BUD2 formulation (Table 3). However, no significant statistical differences were found between the two formulations (*P* > 0.05).

**Table 3:** Characterisation results of the 3D printed suppositories (mean ± SD). *PDI, polydispersity index; ζ, zeta.*

Formulation	Disintegration time (min)	Self-emulsification time (min)	Droplet size (nm)	PDI	ζ Potential (mV)
TOF10-BUD4	7.48 ± 0.44	3.10 ± 0.37	157.47 ± 1.46	0.38 ± 0.04	−30.27 ± 1.02⁎
TOF5-BUD2	7.69 ± 0.30	3.19 ± 0.40	159.37 ± 2.39	0.40 ± 0.01	−35.30 ± 1.51⁎

⁎ Indicates statistically significant difference.

The evaluation of self-emulsification time (i.e., the time taken for droplets of the SEDDS to form a clear solution in water) showed no statistically significant differences between the two formulations (*P* > 0.05). Emulsification is an important parameter in the optimisation of SEDDS, indicating the time taken for the SEDDS to form an emulsion following administration. SEDDS should disperse quickly to form a fine o/w emulsion within the gastrointestinal (GI) tract to release the drugs so that they can exert their clinical effects. Compared to the suppositories previously reported by Seoane-Viaño et al. (Seoane-Viaño et al., 2020b), the current suppositories displayed much faster disintegration times, which can be attributed to the addition of the N,N-DMA. As both tofacitinib citrate (Therapeutic Goods Aministration - Australian Government, 2019) and budesonide (Ali et al., 2010) suffer from poor water solubility, this provides another advantage for their delivery using this innovative approach, potentially improving the clinical effect elicited compared to that of rectal foam and enemas, or oral dosages.

**Fig. 6:**
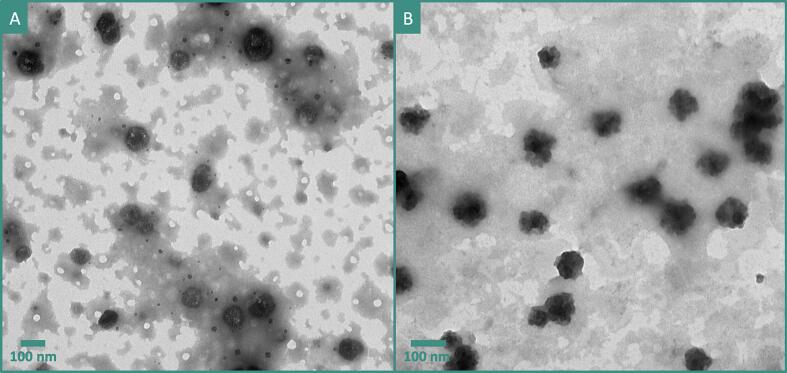
Representative TEM microphotographs of lipid droplets formed following the emulsification of the (A) TOF10-BUD4 and (B) TOF5-BUD2 suppositories, fixed with phosphotungstic acid. The black spots are traces of the phosphotungstic stain.

To characterise the emulsions formed by the suppositories, their droplet size, PDI, and ζ potential were examined (Table 3). The average droplet size of suppositories was found to be 157.47 ± 1.46 and 159.37  ± 2.39 nm for the TOF10-BUD4 and TOF5-BUD2 formulations, respectively (*P* > 0.05). SEDDS typically form an emulsion with droplet sizes ranging between 100 nm and 300 nm (Neslihan Gursoy and Benita, 2004), indicating that the current suppositories have been able to demonstrate appropriate droplet sizes for SEDDS. Whilst Gelucire® 44/14 typically forms microemulsions in aqueous media (Gattefossé, 2022), its formation of larger particle sizes has been previously attributed to the use of coconut oil as a plasticiser in the formulation (Seoane-Viaño et al., 2020b). The PDIs of the TOF10-BUD4 and TOF5-BUD2 formulations were 0.38 ± 0.04 and 0.40 ± 0.01, respectively, with no significant statistical differences (*P* > 0.05). These values indicate that there is heterogeneous dispersion of the particles within the emulsion, however as they are not >0.7, this suggests the absence of a very broad distribution (Danaei et al., 2018). Corroborating with the dynamic light scattering data, the TEM images showed that the formed emulsions were spherical in shape and their sizes were consistent (Fig. 6).

ζ potentials were found to average at −30.27 ± 1.02 mV for the TOF10-BUD4 formulation and − 35.30 ± 1.51 mV for the TOF5-BUD2 formulation, demonstrating the production of emulsions with negatively charged particles (Table 3). ζ potential is an indication of the surface potential and electrostatic repulsion between adjacent particles within a dispersion, evaluating the stability of the emulsion formed. Regardless of the charge, a ζ potential of >30 mV is considered to produce a moderately stable emulsion due to sufficient repulsive forces, limiting the extent of aggregation and flocculation within the dispersion (Joseph and Singhvi, 2019). However, in this case a significant statistical difference (*P* < 0.05) was found between the two formulations, with the TOF5-BUD2 formulation being more stable due to its larger value. Nonetheless, both formulations produced a ζ potential of >30 mV, indicating sufficient emulsion stability. A value lower than this would result in instability due to aggregation and flocculation of the lipid droplets. Compared to the suppositories previously described by Seoane-Viaño et al. (Seoane-Viaño et al., 2020b), the emulsions formed by the suppositories described herein are considered more stable as they possess a more negative ζ potential.

**Fig. 7:**
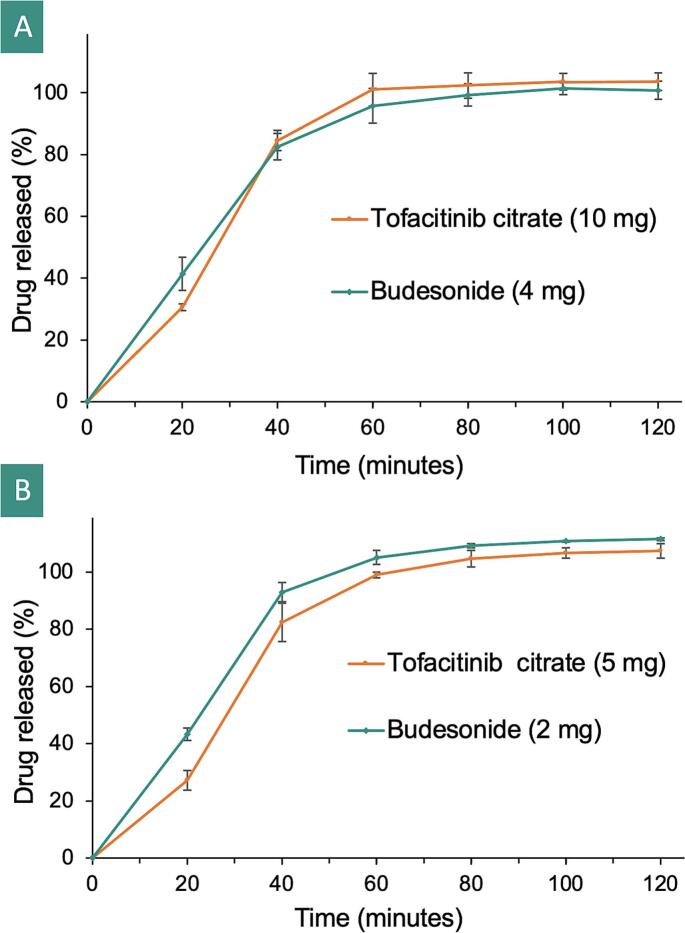
Drug release profiles from the (A) TOF10-BUD4 and (B) TOF5-BUD2 suppositories (*n* *=* *3*) in 0.1 M phosphate buffer (pH 8.0).

*In vitro* drug release was performed to determine the time taken for the full dissolution of the APIs to occur, giving an insight into how the suppositories would behave *in vivo*. Both suppositories release >50% of both tofacitinib citrate and budesonide after 40 min of dissolution, with complete drug dissolution being achieved after ∼80 min (Fig. 7). Tofacitinib citrate exhibited a similar drug release profile from both suppository formulations, with an ƒ_2_ similarity value of 76 obtained from comparing both formulations. Budesonide on the other hand, displayed a faster release rate from the TOF5-BUD2 suppositories compared to the TOF10-BUD4 suppositories, wherein an ƒ_2_ similarity value of 52 was obtained. Whilst budesonide's release profiles were less similar than those of tofacitinib citrate, as both ƒ_2_ similarity values fall between 50 and 100 (Xie et al., 2015), the release characteristics from both formulations were still considered similar. This was further confirmed through statistical analysis, where no significant differences (*P* > 0.05) were observed between the two formulations. These results can be attributed to the low drug percentages within both formulations, resulting in insignificant changes in the suppositories' dissolution and disintegration properties.

An important consideration with suppositories is that they must melt at body temperature (∼37 °C) to release drugs locally (Orlova and Pankrusheva, 2010; Yarnykh et al., 2011). Compared with polyethylene glycol (PEG) based suppositories, which are commonly used as suppository bases in extemporaneous compounding (Jannin et al., 2014), the suppository base described herein is superior due to its lower melting point. In fact, PEG melting points can reach up to 62 °C, limiting or slowing down their melting *in vivo*, which consequently affects the drug release (Paberit et al., 2020). Additionally, PEGs have been associated with local irritation and mucosal damage following administration (Gugulothu et al., 2010). This suggests a benefit of the chosen suppository composition, as not only is it able to be produced by 3D printing instead of manual extemporaneous compounding, reducing human errors and enabling personalised dosing, but also has a melting temperature range more suitable for rectal drug delivery. Furthermore, Gelucire® 44/14 has been used in several studies due to its ability to naturally form a SEDDS (da Fonseca Antunes et al., 2013; Kale and Patravale, 2008; Venkata Raman Kallakunta et al., 2013), improving the bioavailability of poorly water-soluble drugs (Park et al., 2020).

Typically, the use of fusion moulding for the preparation of suppositories is associated with several disadvantages. As an example, because suppository bases have to be vertically poured into moulds in their molten form, there is a risk for the API to sediment down during the solidification process, resulting in its non-uniform distribution within the suppository (Kalmár et al., 2014). This is particularly problematic when a suppository must be split to obtain a smaller drug dose, leading to potential dosing errors (Kaneria et al., 2022). Another drawback of this production method is the need for moulds, restricting the sizes (i.e., the smallest suppository that can be produced weighs 1 g) and shapes in which suppositories can be fabricated in (Jannin et al., 2014). Additionally, moulds are known to have a limited life span, requiring constant replacement and making the process costly. 3D printing on the other hand is a digitised process that enables the design and creation of suppositories in different sizes, shapes and drug doses, all based on various virtual 3D designs (Awad et al., 2021b; Trenfield et al., 2022). Furthermore, since it is an additive manufacturing process that follows a layer-by-layer fashion, this ensures an even spatial distribution of the APIs across the various layers of a suppository (Awad et al., 2023; Andreadis et al., 2022; Awad et al., 2021a).

## 4 Conclusions

For the first time, this work has demonstrated the feasibility of using SSE 3D printing for the fabrication of suppositories loaded with two anti-inflammatory agents aimed at the treatment of ASUC. Despite the TOF10-BUD4 formulation having a faster disintegration and self-emulsification time compared to the TOF5-BUD2 formulation, the differences between the formulations were not statistically significant. In terms of drug release properties, both formulations have been shown to release the APIs in a similar manner despite doubling the drugs' concentrations. This suggests that following administration to the rectum, precise doses of the drugs can be administered locally and released in a timely manner. In spite of the formulations having significantly different ζ-potentials (*P* < 0.05), both formulations have been shown to produce stable emulsions (> 30 mV), improving the solubility of both APIs. Therefore, this work offers a new on-demand manufacturing process for producing suppositories with personalised dosing for individuals suffering from ASUC. This combination treatment approach may provide a fail-safe mechanism if the patient is unresponsive to steroid treatment, which is a common phenomenon in this disorder. Being able to effectively manage this condition not only benefits the patient, but also the health services by reducing the overall cost of treatment.

## Funding

This project has received funding from the Interreg 2 Seas programme 2014–2020 co-funded by the 10.13039/501100008530European Regional Development Fund under subsidy contract “Site Drug 2S07-033”.

## Declaration of Competing Interest

Abdul W. Basit and Alvaro Goyanes are founders of the pharmaceutical company FabRx.

## Data Availability

No data was used for the research described in the article.

## Appendix A Supplementary data

Supplementary material Image 1
