# Supplementary material for: 3D printed multi-drug-loaded suppositories for acute severe ulcerative colitis
Source: Int J Pharm X. 2023 Jan 29;5:100165. doi: 10.1016/j.ijpx.2023.100165 (PMC9982042; doi:10.1016/j.ijpx.2023.100165)
Supplement: Supplementary file 1 — Supplementary material [file mmc1.docx]

Supplementary Materials for:

**3D printed multi-drug-loaded suppositories for acute severe ulcerative colitis**

Atheer Awad^1*^, Eleanor Hollis^1^, Alvaro Goyanes^1,2,3^, Mine Orlu^1^, Simon Gaisford^1^, Abdul W Basit^1,2^

^1^Department of Pharmaceutics, UCL School of Pharmacy, University College London, 29-39 Brunswick Square, London, WC1N 1AX, UK

^2^FabRx Ltd., Henwood House, Henwood, Ashford, Kent, TN24 8DH, UK

^3^Departamento de Farmacología, Farmacia y Tecnología Farmacéutica, I+D Farma (GI-1645), Facultad de Farmacia, and Health Research Institute of Santiago de Compostela (IDIS), Universidade de Santiago de Compostela, 15782 Santiago de Compostela, Spain

*Correspondence:

Dr Atheer Awad

Department of Pharmaceutics, UCL School of Pharmacy, University College London 29-39 Brunswick Square, London, WC1N 1AX, UK

atheer.awad.15@ucl.ac.uk

**S1.1 Thermogravimetric analysis (TGA)**

Thermogravimetric analysis (TGA) was performed to assess the thermal stability of budesonide and tofacitinib citrate. Average samples of 8–10 mg of the raw drug powders were placed in open aluminium pans and heated at a temperature range between 50 °C to 300 °C. Heating was performed at a rate of 10 °C /min using a Discovery TGA (TA instruments—Waters LLC, New Castle, DE, USA). Nitrogen was used as a purge gas at a flow rate of 25 mL/min. Data were collected and analysed using TA Instruments Trios software (Version 4.5.0.5), where the percentage mass loss of each drug was calculated with respect to the change in temperature.


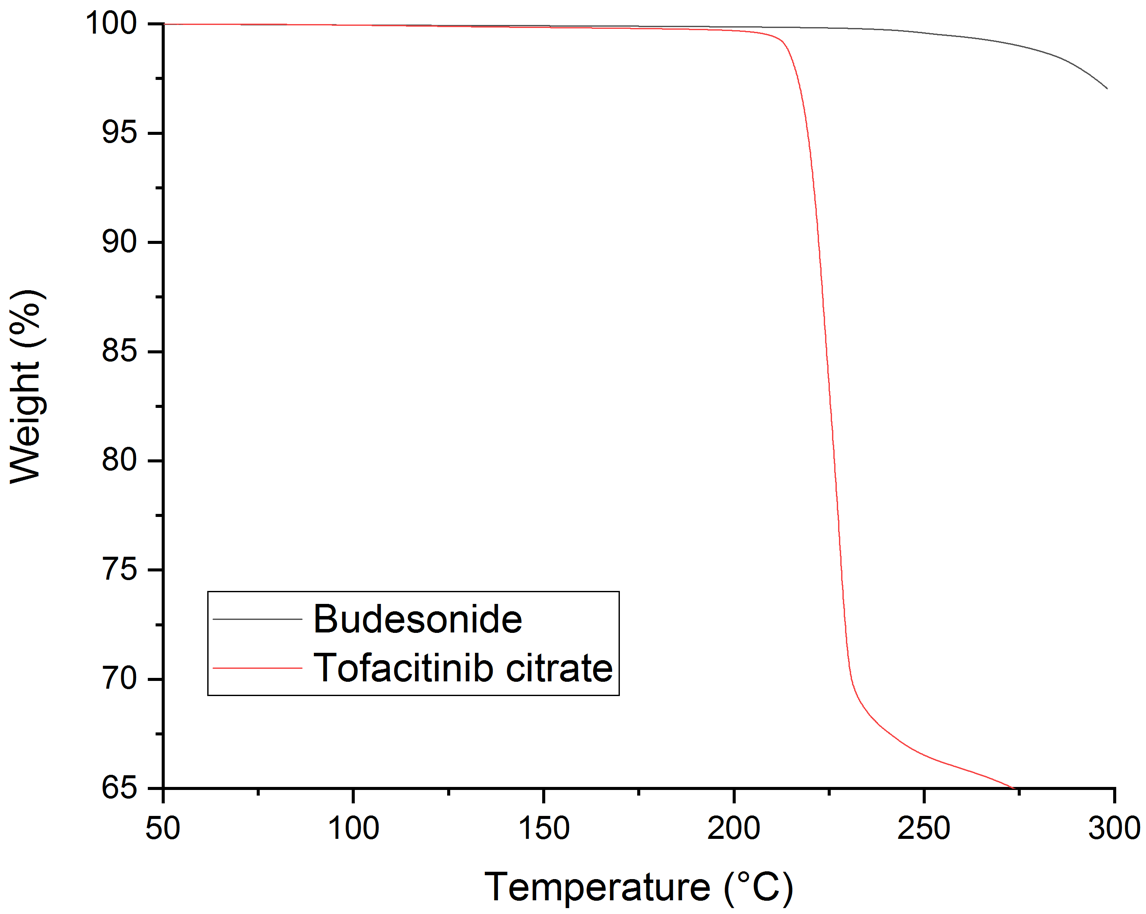


**Figure S1.** Thermogravimetric analysis (TGA) results of the raw budesonide and tofacitinib citrate powders.
